# Donkey Orchid Symptomless Virus: A Viral ‘Platypus’ from Australian Terrestrial Orchids 

**DOI:** 10.1371/journal.pone.0079587

**Published:** 2013-11-05

**Authors:** Stephen J. Wylie, Hua Li, Michael G. K. Jones

**Affiliations:** Australian Plant Virology Laboratory, Western Australian State Agricultural Biotechnology Centre, School of Veterinary and Life Sciences, Murdoch University, Perth, Australia; University of California, San Francisco, United States of America

## Abstract

Complete and partial genome sequences of two isolates of an unusual new plant virus, designated Donkey orchid symptomless virus (DOSV) were identified using a high-throughput sequencing approach. The virus was identified from asymptomatic plants of Australian terrestrial orchid *Diuris longifolia* (Common donkey orchid) growing in a remnant forest patch near Perth, western Australia. DOSV was identified from two *D. longifolia* plants of 264 tested, and from at least one plant of 129 *Caladenia latifolia* (pink fairy orchid) plants tested. Phylogenetic analysis of the genome revealed open reading frames (ORF) encoding seven putative proteins of apparently disparate origins. A 69-kDa protein (ORF1) that overlapped the replicase shared low identity with MPs of plant tymoviruses (*Tymoviridae*). A 157-kDa replicase (ORF2) and 22-kDa coat protein (ORF4) shared 32% and 40% amino acid identity, respectively, with homologous proteins encoded by members of the plant virus family *Alphaflexiviridae*. A 44-kDa protein (ORF3) shared low identity with myosin and an autophagy protein from Squirrelpox virus. A 27-kDa protein (ORF5) shared no identity with described proteins. A 14-kDa protein (ORF6) shared limited sequence identity (26%) over a limited region of the envelope glycoprotein precursor of mammal-infecting Crimea-Congo hemorrhagic fever virus (*Bunyaviridae*). The putative 25-kDa movement protein (MP) (ORF7) shared limited (27%) identity with 3A-like MPs of members of the plant-infecting *Tombusviridae* and *Virgaviridae*. Transmissibility was shown when DOSV systemically infected *Nicotiana benthamiana* plants. Structure and organization of the domains within the putative replicase of DOSV suggests a common evolutionary origin with ‘potexvirus-like’ replicases of viruses within the *Alphaflexiviridae* and *Tymoviridae*, and the CP appears to be ancestral to CPs of allexiviruses (*Alphaflexiviridae*). The MP shares an evolutionary history with MPs of dianthoviruses, but the other putative proteins are distant from plant viruses. DOSV is not readily classified in current lower order virus taxa.

## Introduction

The study of plant viruses has focused primarily on domesticated plants displaying visible signs of infection. This has lead to a misapprehension that most viruses induce symptoms in their hosts. Recent utilization of high-throughput nucleotide (nt) sequencing technologies to analyse RNA from apparently healthy wild plants has revealed an abundance of RNA viruses in them [1,2,3,4,5,6] demonstrating that symptomless virus infection may represent the normal condition in wild plants. 

The Southwest Australian Floristic Region occupies some 300,000 km^2^. It is a region of high biodiversity that is geographically and biologically isolated by vast deserts to the northeast and east, and oceans to the south and west. Its approximately 8000 indigenous vascular plant species are adapted to an unpredictable Mediterranean-type climate, fire, and some of the world’s most ancient and least fertile soils [7]. Amongst them are 394 terrestrial orchid species (family *Orchidaceae*) belonging to 28 genera [8]. All but one of these exist as leafy plants for only a few weeks a year; for the remainder they are underground tubers. Seventy-six species have a high level of conservation risk [9]. Many are threatened through the direct or indirect actions of humans, but intrinsic factors in their biology such as their modes of pollination by deceiving insects using sex pheromone mimicry and physical mimicry, low rates of fruit set, mycorrhizal specificity, and habitat specialization also play roles in species rarity [10,11,12]. The genus *Diuris* Sm. is named after the twin lateral sepals on the flowers, although the distinctive ear-like petals have given them their common name of Donkey Orchid. All but one of the 50 *Diuris* species are endemic to Australia. The genus *Caladenia* R.Br. (spider orchids) contains about 240 species, the majority of which are endemic to Australia [8,12]. 

Indigenous viruses that infect the Western Australian flora are largely unstudied. The few described from the region are potyviruses (family *Potyviridae*), various betaflexiviruses (*Betaflexiviridae*), poacevirus (*Potyviridae*), and an unassigned member of the *Secoviridae* [4,5,6,13,14]. Australian terrestrial orchids may be widely infected with both exotic and indigenous viruses. Eight virus species: two capillo-like viruses, one partitivirus, one polerovirus, four potyviruses, were recently described from eight plants belonging to four *Diuris* species [6]. In another study, plants of two *Caladenia* species, and a hammer orchid (*Drakaea elastica* Lindl.) were infected with a new poacevirus, the first described from a non-poaceous host [5].

Here, we describe a complete and a near-complete genome sequence from two isolates of an unusual virus, provisionally named Donkey orchid symptomless virus, from wild plants of common donkey orchid (*Diuris longifolia* R.Br), and confirm its presence in wild pink fairy orchid (*Caladenia latifolia* R.Br) in Western Australia. The genome organization and identities of the putative gene products of the new virus are discussed in relation to those of previously described viruses. 

## Materials and Methods

### Ethics Statement

Orchid leaf samples were collected in 2011, 2012 and 2013 under Western Australian Department of Environment and Conservation flora licenses.

### Plants

Leaf material (~100 mg per plant) was taken in 2011 at random from 31 individuals of a scattered population of *D. longifolia* plants growing in a patch of remnant forest known as Caporn Park (GPS location -31.732438, 115.806838) in the locality of Mariginiup, Western Australia. After RNA extraction, remaining leaf material was lyophilized and stored at -20°C. These plants were analysed using a high-throughput sequencing approach as described below. Later, an incidence survey was carried out using 233 *D. longifolia* plants and 129 *C. latifolia* plants, as described below.

Both lyophilized and fresh orchid leaf material was used for manual inoculation to indicator plants. Leaf material was collected from the two wild orchid plants in Caporn Park that were the original source of virus isolates. Orchid leaf material was ground in 0.1M phosphate buffer (pH 7.0) and manually applied with diatomaceous earth to two to four each of *Nicotiana benthamiana*, *N. glutinosa*, *Chenopodium quinoa* and *C. amaranticolor* seedlings. An equal number of plants were mock inoculated. Plants were examined for symptoms of infection weekly for 6 weeks following inoculation, and young, un-inoculated leaves were tested for the presence of DOSV at week 6 by RT-PCR assay as described below. 

### RNA extraction and Illumina sequencing

Total RNA, including dsRNA, was extracted from thirty-one 50 mg samples of *D. longifolia* leaf material using the Powerplant® RNA isolation kit (Mo Bio Laboratories). cDNA was synthesized from dsRNA using random 12-mer primers that had a 16 nt adaptor at the 5’ end. Distinct four-nucleotide tags were added to cDNAs derived from each of the 31 samples during PCR amplification with barcoded primers that annealed to the 16 nt adapter sequence added during cDNA synthesis. Amplification conditions were as follows: five cycles of 95°C for 20 s, 25 °C for 20 s, 72°C for 20 s, followed by 35 cycles of 95°C for 20 s, 40 °C for 20 s, 72°C for 20 s. The resulting amplicons were separated on a 1 % agarose gel and fragments in the 200-600 nt range were purified using Minelute (Qiagen) columns. Samples were quantified on a spectrometer and 0.5 μg of RT-PCR product representing each sample was pooled for sequencing. Library construction and 100 cycle paired-end sequencing on an Illumina HiSeq2000 was done by Macrogen Inc (Seoul, S. Korea). The sequence read data was submitted to the Sequence Read Archive (NCBI) and was granted accession code PSUB001725.

### High-throughput sequence analysis

Joining paired sequences, separating the tagged sequence reads, *de novo* assembly of contigs, editing where required, and calculating genome architecture was done primarily using CLC Genomics Workbench v6.0.5 (CLC Bio) and Geneious Pro v6.1.6 (Biomatters) packages. Parameters for *de novo* assembly of contigs were minimum overlap of 50 % of read length, 10 % maximum gaps per read. Three assemblies were done for each data set using minimum overlap identities of 80 %, 90 %, and 95 %. Consensus sequences were compared with sequences in GenBank using Blastx. Open reading frames and identities of deduced proteins were predicted by identity with annotated virus sequences available on GenBank, at the Conserved Domain Database (CDD) within NCBI, and InterProScan accessed at http://www.ebi.ac.uk/Tools/pfa/iprscan/. Estimates of evolutionary relationships were calculated from global alignments of amino acid sequences within MEGA5 using the statistical methods Neighbor-Joining, Maximum Parsimony, and Maximum Likelihood (ML), and Mr Bayes within Geneious Pro. Internal settings were a cost matrix of 65 %, gap open penalty of 12 and gap extension penalty of 3. Bootstrap analysis [15] of 1,000 replicates was used to assess support for relationships. The ML trees presented were congruent with other analyses. The substitution model used for ML analysis was General Reverse Transcriptase, which had the lowest Bayesian information criterion score, and the ML heuristic method used was Nearest Neighbor Interchange. Transmembrane domains were predicted after analysis with the Tmpred program (http://www.ch.embnet.org/cgi-bin/TMPRED_form_parser). Pairwise identities between sequences were calculated by ClustalW alignment in MEGA5 [16]. Comparison of sequences was done by Blast and within the Pairwise Sequence Comparison (PASC) database for analysis of pairwise identity distribution within viral families (http://www.ncbi.nlm.nih.gov/sutils/pasc/viridty.cgi?textpage=overview). 

Prediction of the secondary structure of 3’UTR regions was done within RNAfold [17] at http://rna.tbi.univie.ac.at/cgi-bin/RNAfold.cgi.

### Confirmation of genome sequence

Amplification using RT-PCR of the genome of isolate Mariginiup11 was done using overlapping primer pairs (Table S1) whose design was based on sequence generated using Illumina technology. The amplicons of ~900 nt overlapped each other by approximately 100 nt. cDNA was generated from total plant RNA using ImProm-II™ reverse transcriptase (Promega) primed with random nonamer primers. PCR conditions were denaturation at 95°C for 10 sec, primer annealing at 60°C for 30 sec, extension at 72°C for 30 sec, over 35 cycles using GoTaq® DNA polymerase (Promega). Automated Sanger sequencing was carried out using an Applied Biosystems/Hitachi 3730 DNA Analyzer with BigDye terminator V3.1 chemistry (AB).

Both the 5’ and 3’ ends of the genome of isolate 11 were confirmed using a 5’ RACE kit, version 2.0 (Invitrogen) following the manufacturer’s procedure. Two virus-specific primers were designed to facilitate amplification of the 5’ region. RACE51 5’-CAAGGAGGTGATTTTCGATG-3’ annealed at nt 356-375. Nested primer RACE52 5’- CATGGCGGTGGAGCTGGGTG-3’ annealed at nt 323-342. Similarly, two specific primers were designed to amplify the 3’ end of the genome. Primer RACE31 5’- CGCAGGGAGTCTGACCTACG-3’ annealed within the MP at nt 7516-7535, and primer RACE32 5’- GAGGGACACCACTCGCAAAT-3’ at nt 7556-7575. 

### Incidence survey

144 leaf samples of *D. longifolia* plants and 102 samples of *C. latifolia* R.Br. (pink fairy orchid) plants were collected in 2011 and 2012 from Caporn Park. A further collection of 89 leaf samples of *D. longifolia* and 27 of *C. latifolia* leaves was carried out at a remnant bushland site at Murdoch University campus in 2012 (GPS location -32.065394, 115.840782). Total plant RNA was extracted from groups of 10-11 leaves using the Powerplant® RNA isolation kit (Mo Bio Laboratories). RT-PCR using two primer pairs was used to detect the virus in orchid leaf samples. The primer pairs used were DOSV8F5200 which anneals at nt 5200-5223 within the 44-kDa protein gene, and DOSV8R6100 which anneals at nt 6080-6100 within the CP gene, and DOSV10F6800 which anneals at nt 6800-6820 within the 27-kDa protein gene and DOSV10R7700 (Table S1) which anneals at nt 7687-7700 in the MP gene. Synthesis of cDNA and its amplification was as above. The same primer pairs and amplification conditions were used to screen experimental host plants for presence of the virus.

## Results

### Sequence assembly

30,007,801 reads of 100 nt each were obtained after Illumina sequencing. After the sequences were sorted by tag identity, contigs were generated by *de novo* assembly and analysed by Blastx. Two closely related but distinct sequences with identity to previously described viruses were obtained, one from each of two *D. longifolia* plants. After some manual annotation, the virus-like sequences were of 7838 nt and 7443 nt. When aligned, the nucleotide sequences shared 92 % pairwise identity. The sequences were designated isolates Mariginiup11 (7838 nt) and Mariginiup12 (7443 nt). The complete genome sequence of isolate Mariginiup11 was assigned GenBank accession code KC923234, and the partial isolate Mariginiup12 was assigned accession code KC923235. The sequence of isolate Mariginiup11 was constructed from 49,005 reads of the 1,201,112 reads (4.07 %) derived from plant Di4. Coverage range was from 9-fold at nt 1-9 to 2041-fold at nt 6738. Mean coverage across the genome was 632-fold (SD = 310). Mean pairwise identity of raw reads was 97.5 %. The sequence of isolate Mariginiup12 was constructed from 32,768 reads of the 977,033 reads (3.35 %) derived from plant Di3. Coverage range was from 8-fold at nt 1-16 to 1875-fold at nt 5072. Mean coverage was 441-fold (SD = 320.5). Mean pairwise identity of raw reads was 96.9 %. The sequence of the 5’ end of the Mariginiup12 genome was not determined. Based on alignment with isolate Mariginiup11, it is estimated to be about 410 nt short of the complete genome sequence. 

### Resequencing

Primers (Table S1) were used to re-amplify the genome of isolate Mariginiup11 from randomly primed cDNA from *D. longifolia* plant Di4. Amplicons were sequenced directly in each direction using the primers that generated them. Primer sequences were removed before a contig was generated. Identity between the original (Illumina) sequence and the Sanger sequence was 99.4 %. The 5’ and 3’ ends of the genome of isolate Mariginiup11 were both determined using a RACE (random amplification of cDNA ends) procedure that confirmed the sequence generated by the Illumina method.

### Genome organization and identity

**Figure 1:**
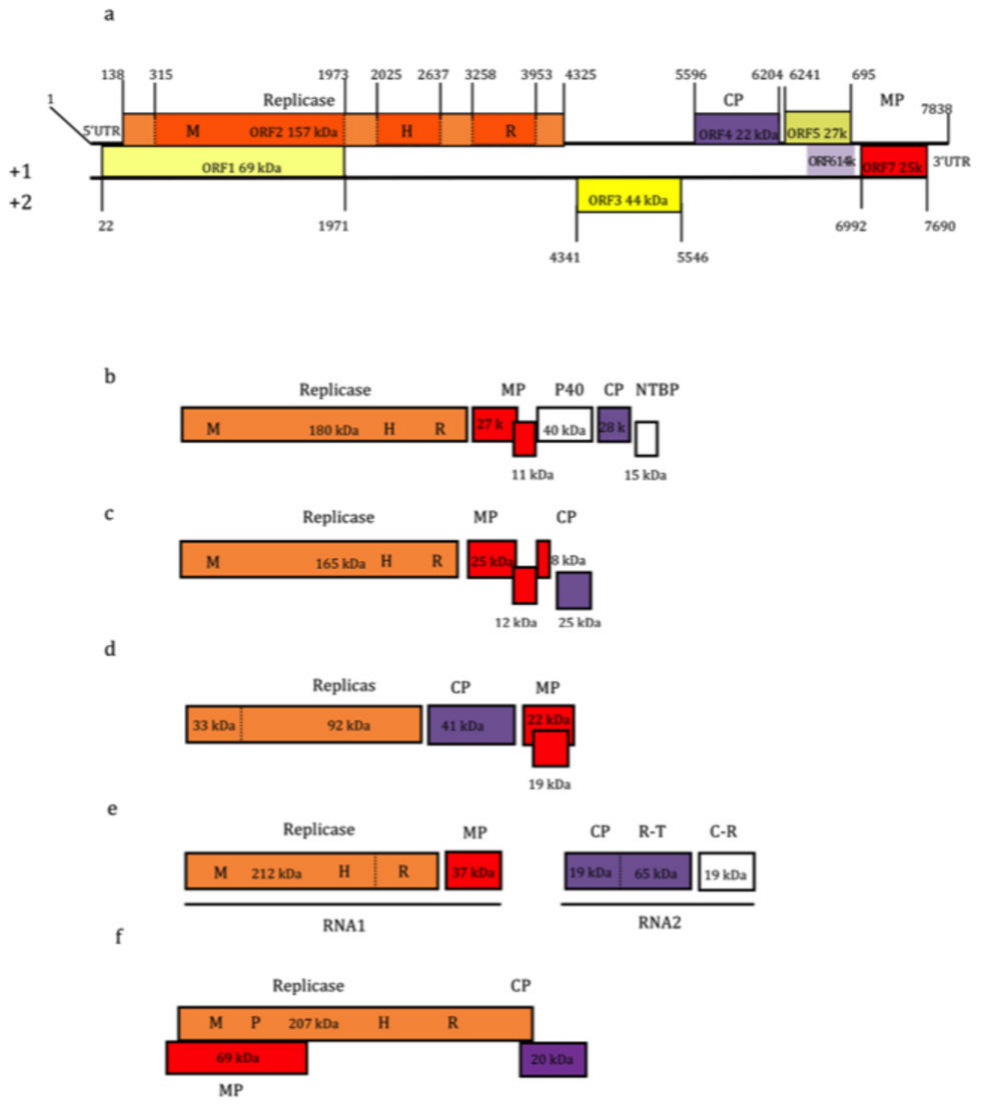
Genome organization of Donkey orchid symptomless virus. Genome organization of Donkey orchid symptomless virus isolate Mariginiup11 (a), showing the nucleotide positions of open reading frames (ORF) including putative coat protein (CP), movement protein (MP), and untranslated regions (UTR), and calculated sizes of putative proteins. Open reading frames were designated with respect to the replicase (ORF1). Diagrams of genome organization of (b) *Garlic* *virus* *A* (family *Alphaflexiviridae*, genus *Allexivirus*) encodes a 40-kDa protein (P40) and nucleotide binding protein (NTBP), (c) *Plantago asiatica* *mosaic* *virus* (*Alphaflexiviridae*, *Potexvirus*), (d) Tomato bushy stunt virus (TBSV) (*Tombusviridae*, *Tombusvirus*), (e) Sorghum chlorotic spot virus (SCSV) (*Tombusviridae*, *Furovirus*), and (f) *Turnip* *yellow* *mosaic* *virus* (*Tymoviridae*, *Tymovirus*) are shown (not drawn to scale). Within the replicase, the papain-like protease (P), methyltransferase (M), helicase (H), and RNA-dependent RNA polymerase (RdRp) domains are indicated where present. In TBSV and SCSV, read-through opal stop codons are indicated by a dotted line, R-T = read-through region, and C-R = cysteine-rich protein.

The genomes of both isolates contained seven open reading frames (ORF) flanked by a 5’ untranslated region (UTR) and 3’ UTR (Figure 1), although this is assumed for isolate Mariginiup12 because the 5’ terminal region of the genome was not determined. A 3’ polyadenylation sequence was not detected for either genome. Neither the 5’UTR nor the 3’UTR shared identity with those of other viruses. The 3’UTR sequences of the two isolates were 146-147 nt in length and shared 91.8 % nt sequence identity. 

### ORF1: 69-kDa protein

ORF1 overlapped ORF2 in frame +1 of isolate Mariginiup11 with respect to the replicase ORF (Figure 1). It extended from nt 22-1974 and encoded a putative protein calculated to have a mass of 69.2-kDa. The N-terminal region of this ORF from isolate Mariginiup12 was not determined. Sequences from both isolates shared 84 % amino acid (93 % nt) identity. The protein is rich in serine, which comprise 20 % of the amino acids. It shared 42 % identity with the 69-kDa MP of *Turnip yellow mosaic virus* (genus *Tymovirus*) (Figure 1), but only over two short regions near the termini (nt 154-324, 1681-1830).

### ORF2: Replicase

**Figure 2:**
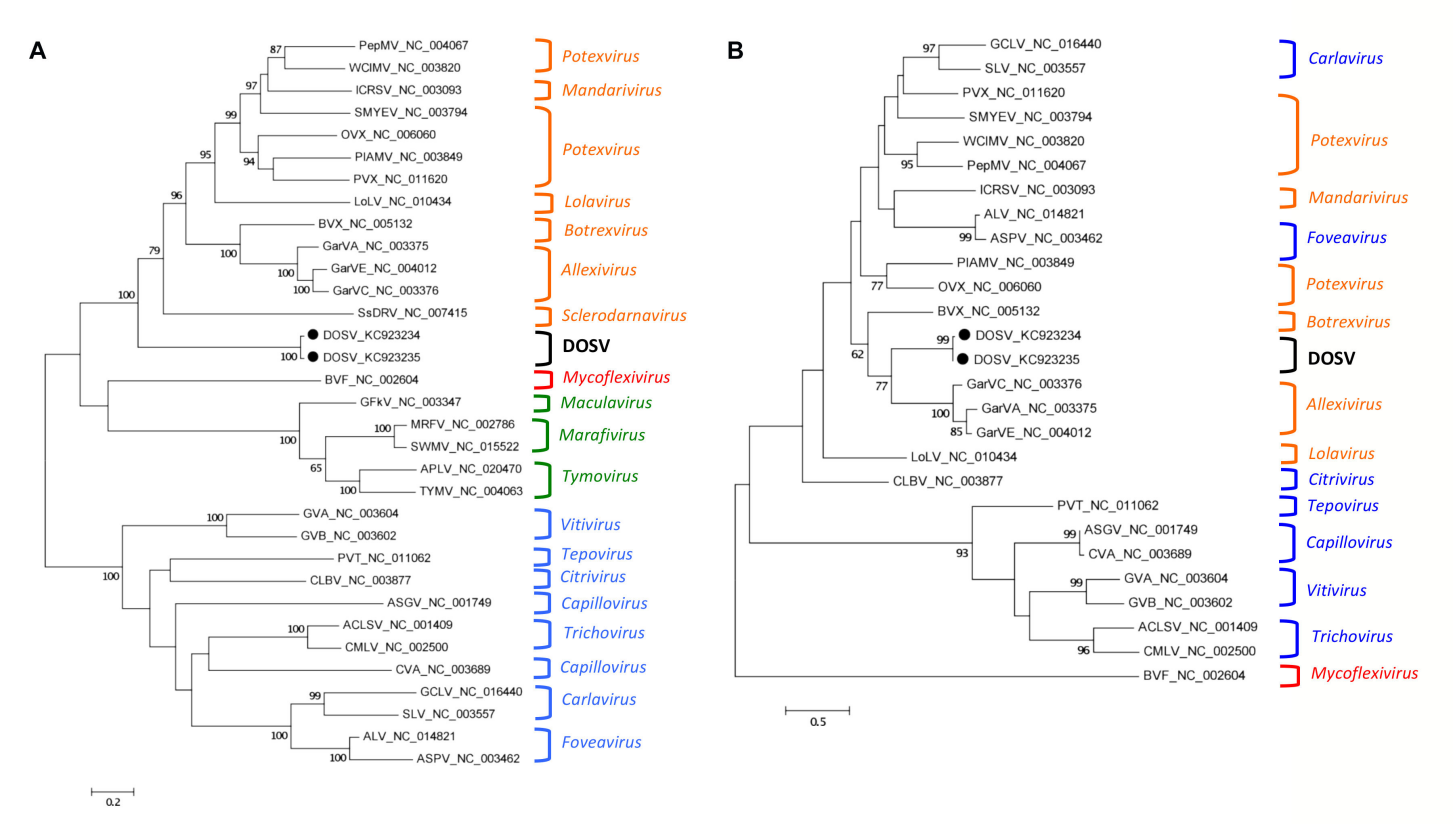
Evolutionary relationships of Donkey orchid symptomless virus replicase and coat protein. Donkey orchid symptomless virus (DOSV) (represented with a black dot) replicase (a), and coat protein (b) using a General Reverse Transcription model with homologous proteins of representative viruses within the order *Tymovirales*. Relationships were inferred for amino acid sequences using the Maximum Likelihood method. The abbreviated names of the viruses used and their GenBank accession codes are shown. Genus names are given on the right. Genus names are colored according to family classification. Orange represents the *Alphaflexiviridae*, red represents the *Gammaflexiviridae*, green represents the *Tymoviridae*, and blue represents the *Betaflexiviridae*. The position of DOSV isolates are shown as black dots. ACLSV, *Apple* *chlorotic* *leaf* *spot* *virus*; ALV, *Apricot* *latent* *virus*; APLV, *Andean* *potato* *latent* *virus*; ASGV, *Apple* *stem* *grooving* *virus*; ASPV, *Apple* *stem* *pitting* *virus*; BVF, *Botrytis virus* *F*; BVX, *Botrytis virus* *X*; CLBV, *Citrus leaf* *blotch* *virus*; CMLV, *Cherry* *mottle* *leaf* *virus*; CVA, *Cherry* *virus* *A*; GarVA, *Garlic* *virus* *A*; GarVC, *Garlic* *virus* *C*; GarVE, *Garlic* *virus* *E*; GCLV, *Garlic* *common* *latent* *virus*; GFkV, *Grapevine* *fleck* *virus*; GVA, *Grapevine* *virus* *A*; GVB, *Grapevine* *virus* *B*; ICRSV, *Indian* *citrus* *ringspot* *virus*; LoLV, *Lolium latent* *virus*; MRFV, *Maize* *rayado* *fino* *virus*; OVX, *Opuntia virus* *X*; PepMV, *Pepino* *mosaic* *virus*; PlAMV, *Plantago asiatica* *mosaic* *virus*; PVT, *Potato* *virus* *T*; PVX, *Potato* *virus* *X*; SLV, *Shallot* *latent* *virus*; SMYEV, *Strawberry* *mild* *yellow* *edge* *virus*; SsDRV, *Sclerotinia sclerotiorum* *debilitation-associated* *RNA* *virus*; SWMV, *Switchgrass* *mosaic* *virus*; TYMV, *Turnip* *yellow* *mosaic* *virus*; WClMV, *White* *clover* *mosaic* *virus*. The percentage of replicate trees above 60 % in which the associated taxa clustered together in the bootstrap test (1000 replicates) is shown next to the branches. Units are amino acid substitutions per site.

The complete putative viral replicase from isolate Mariginiup11 was 4188 nt in length, and is calculated to encode a protein of 157.6-kDa. The N-terminus of the replicase was not obtained for isolate Mariginiup12. Aligned, the replicases of the two isolates shared 95.5 % amino acid (91.5 % nt) identity over the common region. The complete amino acid sequence of the replicase of isolate Mariginiup11 shared highest identity (31-32 %) with replicase sequences of members of the family *Alphaflexiviridae* (Table 1). Its sequence fits with high confidence between members of the *Alphaflexiviridae* and a group formed by members of the *Gammaflexiviridae* and the *Tymoviridae* (Figure 2a). CDD and InterProScan [18 predicted the presence of three active domains within the replicase: a viral methyltransferase (Met), a helicase, and an RNA-dependent RNA polymerase (RdRp), typical of some flexivirus genomes (Figure 1). The putative Met domain was approximately 1659 nt in length. The conserved Met motif DEAD [19] was present at its C-terminal region from nt 1944-1955. A helicase domain estimated to be 613 nt long contained conserved motifs A (GKS) and B (DE) [20], which were identified from nt positions 2040-2048 and 2217–2222, respectively. The putative RdRp domain estimated to be 696 nt in length contained the conserved core motif S/TG (X_3_)T(X_3_)NS/T(X_22_)GDD (where X is any amino acid residue) [21] at nt 3690-3797. 

**Table 1:** Description of Donkey orchid symptomless virus open reading frames and putative gene products with matches to proteins of other organisms.

**ORF**	**Location (nt)^a^**	**MW (kDa)**	**Context start codon^b^**	**Stop codon^c^**	**Match: species**	**Match: higher classification^d^**	**Host/origin**	**Protein match**	**Protein Accession code**	**Query cover (%)**	**E-value**	**Max amino acid identity (%)**
ORF1 69-kDa protein	22-1974	69.1	GACGAUGCGG	UGA	*Turnip yellow mosaic virus*	*Tymoviridae, Tymovirus*	Turnip	Movement protein	NC_004063	20	0.092	42
	(1-1575)	-	-	(UGA)								
ORF2 Replicase	138-4325	157.6	UGUCAUGCUA	UAA	*Plantago asiatica mosaic virus*	*Alphaflexiviridae, Potexvirus*	*Plantago asiatica*	Replicase	BAG1]43	95	0.0	31
	(1-3932)			(UAA)	*Strawberry mild yellow edge virus*	*Alphaflexiviridae, Potexvirus*	Strawberry	Replicase	CAE12227	93	0.0	32
					*White clover mosaic*	*Alphaflexiviridae, Potexvirus*	White clover	Replicase	BAK78865	94	0.0	31
					*Turnip yellow mosaic virus*	*Tymoviridae, Tymovirus*	Turnip	Replicase	NC_004063	74	9e-35	46
ORF3 44-kDa protein	4341-5543	43.9	CCCUAUGCCGC	UAA	*Squirrelpox virus*	*Poxviridae, Chordopoxvirinae*	Red Squirrel	Autophagy protein 16	ABD51465	45	1e-11	22
	(3945-5150)	44.5	(UCCCAUGUCAC)	(UAA)								
					*Salpingoeca rosetta*	*Salpingoecidae, Salpingoeca*		Myosin tail	EGD73038	46	3e-17	20
ORF4 Coat protein	5596-6204	22.1	CCUUAUGUCGA	UAA	*Garlic virus A*	*Alphaflexiviridae, Allexivirus*	Garlic	Coat protein	NP_569130	88	1e-33	40
	(5200-5808)	22.1	(UUCCAUGUCGA)	(UAA)	*Garlic virus C*	*Alphaflexiviridae, Allexivirus*	Garlic	Coat protein	NP_569136	96	3e-33	37
					*Garlic virus X*	*Alphaflexiviridae, Allexivirus*	Garlic	Coat protein	CAC83703	93	5e-33	39
ORF5 27-kDa protein	6241-6954	27.5	GAUCAUGCUCC	UGA	No matches							
	(5845-6561)	27.6	(AAUCAUGCUCC)	(UGA)								
ORF6 14-kDa protein	6602-6985	13.6	CAUCAUGAACU	UGA	*Crimea-Congo hemorrhagic fever virus*	*Bunyaviridae, Nairovirus,*	Human	Envelope glycoprotein precursor	AAA86616	92	0.056	26
	(6206-6589)		(CAUCAUGAACU)	(UGA)					NP_950235			
ORF7 Movement protein	6992-7690	22.5	UCCAAUGGCCA	UGA	*Sweet clover necrotic mosaic virus*	*Tombusviridae, Dianthovirus*	Sweet clover	3a movement protein	NP_620676	86	4e-13	26
	(6596-7297)	22.5	(UCCAAUGGCGA)	(UGA)	*Sorghum chlorotic spot virus*	*Virgaviridae, Furovirus*	Sorghum	3a movement protein	NP_659021	77	1e-12	27
					*Carnation ringspot virus*	*Tombusviridae, Dianthovirus*	Carnation	3a movement protein	NP_613257	96	2e-12	27

a Location of genes on genome of isolate Mariginiup11 (top) and Mariginiup12 (below in parentheses), where known

b Context of start codon of isolate Mariginiup11 (top) and Mariginiup12 (below in parentheses), where known.

c Stop codon for isolates Mariginiup11 and Mariginiup 12 (below in parentheses).

d Family, genus

### ORF3: 44-kDa protein

An ORF of 1203 nt was predicted in frame +2 between the replicase and coat protein (CP) genes (Figure 1). Positions in isolate Mariginiup11 were nt 4341-5543, and in isolate Mariginiup12 3945-5147. The ORF is calculated to encode a protein of 43.9-kDa. ORF3 shared 88 % amino acid (89 % nt) identity between the two isolates. Analysis with CDD and Interproscan identified two possible domains. A signal peptide-like domain was identified at the N-terminus at residues 1-19 of isolate Mariginiup11 (nt 4355-4411) and 1-24 of isolate Mariginiup12 (nt 3945-4016). The second domain (nt 4841-5233 in isolate Mariginiup11) shared up to 24 % amino acid identity with autophagy protein 16 from Squirrelpox virus (Table 1). Analysis with Interproscan predicted three coiled-coil motifs, located at residues 166-187, 194-215, and 271-292 in isolate Mariginiup11. Coiled coils are structural motifs in proteins in which 2-7 alpha-helices are coiled together. Coiled coil type proteins are involved in gene regulation, e.g. transcription factors. 

### ORF4: Coat protein gene

An ORF of 609 nt encoding a putative coat protein (CP) of 22-kDa was present at nt 5596-6204 in isolate Mariginiup11 and at nt 5200-5808 in isolate Mariginiup12 (Figure 1). The CP sequences shared 99 % amino acid (92 % nt) sequence identities between the two isolates. The amino acid sequence shared greatest identity (39-40 %) with the CP protein sequences of species of *Allium*-infecting allexiviruses (family *Alphaflexiviridae*) (Table 1), and phylogenetic analysis using several statistical methods showed with high confidence that it may be ancestral to them (Figure 2b). 

### ORF5: 27-kDa protein gene

An ORF calculated to encode a protein of 27.5-kDa was from nt 6241-6954 in isolate Mariginiup11 and nt 5845-6561 in isolate Mariginiup12. It lay between the putative CP and MP genes (Figure 1). The protein sequence of the two isolates shared 94 % amino acid (92 % nt) sequence identity. The amino acid sequence had no apparent amino acid sequence homology with known proteins, and no active domains or structural motifs were identified. 

### ORF6: 14-kDa protein

A small ORF overlapped ORF5 in frame +1. It is calculated to encode a protein of 13.6-kDa. Its greatest amino acid identity (26 %) was to the N-terminal region of the envelope glycoprotein precursor of Crimea-Congo hemorrhagic fever virus (*Bunyaviridae*) (Table 1). 

### ORF7: Movement protein gene

**Figure 3:**
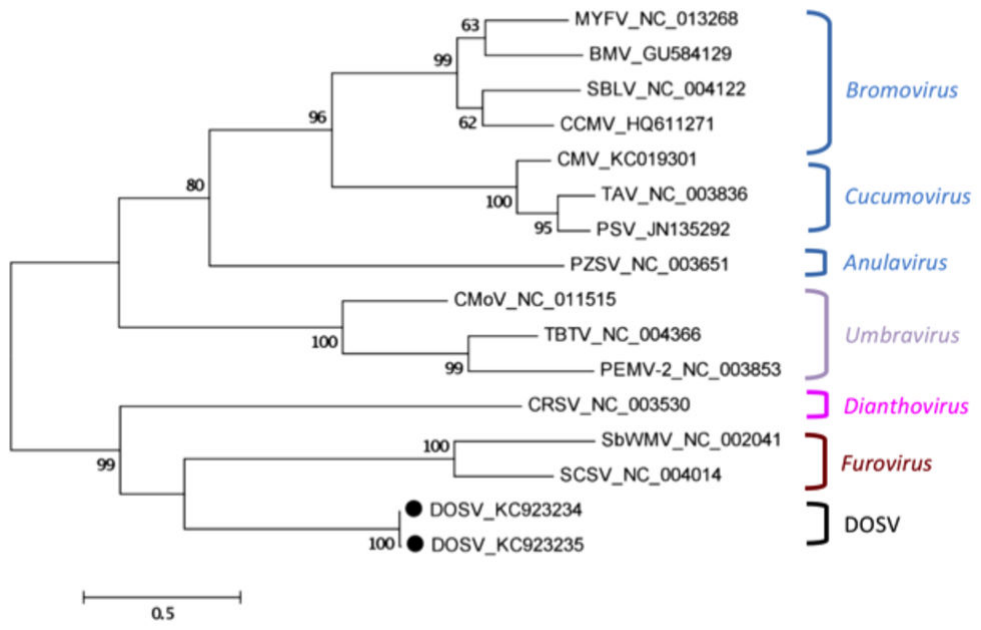
Evolutionary relationships of Donkey orchid symptomless virus movement protein. The movement protein of Donkey orchid symptomless virus (DOSV) isolates (represented by black dots) compared with representative viruses that have a 3A-like movement protein. Phylogeny is inferred with the Maximum Likelihood method using a General Reverse Transcription model. Abbreviated virus names and their GenBank accession codes are shown. Genus names are given on the right. Blue represents the family *Bromoviridae*, purple represents the genus *Umbravirus* (family unassigned), brown represents the *Virgaviridae*, and pink represents the *Tombusviridae*. TAV, *Tomato* *aspermy* *virus*; PSV, *Peanut* *stunt* *virus*; CMV, *Cucumber* *mosaic* *virus*; MYFV, *Melandrium yellow* *fleck* *virus*; BMV, *Brome* *mosaic* *virus*; SBLV, *Spring* *beauty* *latent* *virus*; CCMV, *Cowpea* *chlorotic* *mottle* *virus*; TBTV, *Tobacco* *bushy* *top* *virus*; PEMV-2, *Pea* *enation* *mosaic* *virus-2*; CMoV, *Carrot* *mottle* *virus*; SbWMV, *Soil-borne* *wheat* *mosaic* *virus*; SCSV, *Sorghum chlorotic* *spot* *virus*; PZSV, *Pelargonium zonate* *spot* *virus*; CRSV, *Carnation* *ringspot* *virus*. The percentage of replicate trees above 60 % in which the associated taxa clustered together in the bootstrap test (1000 replicates) is shown next to the branches. Units are amino acid substitutions per site.

The ORF closest to the 3’ end of the genome occurred from nt 6992-7690 in frame +1 in isolate Mariginiup 11 and nt 6596-7294 in isolate Mariginiup12 (Figure 1). It was calculated to encode a protein of 25.5-kDa. The proteins of the two isolates shared 98 % amino acid (93 % nt) sequence identity. Its nt sequence shared 26-27 % amino acid identity with the 34-kDa 3A-like movement proteins (MP) of RNA2 of dianthoviruses (family *Tombusviridae*) such as *Carnation ringspot virus*, and with 37-kDa MPs of RNA1 of furoviruses (family *Virgaviridae*) such as *Sorghum chlorotic spot virus* (Table 1). It is less closely related to 3A-like MPs of umbraviruses and members of the family *Bromoviridae* (Figure 3). Tmpred predicted a transmembrane region at amino acid residues 65–84 (nt 7183-7242 in isolate Mariginiup11, nt 6788-6847 isolate Mariginiup12) that is consistent with its putative role as a movement protein. 

Non-polyadenylated viruses usually have a transfer RNA-like (tRNA) secondary structure within the 3’UTR. A pseudoknot structure was predicted using RNAfold from the sequence of the 3’UTR region of both isolates (Figure S1). The structure predicted was calculated to have a minimum free energy of -46.70 kcal/mol at 37°C.

### Inoculation of experimental host plants

Homogenates of lyophilised and fresh leaves of *D. longifolia* plants Di3 and Di4 were manually inoculated to 10 each of young seedlings of *Nicotiana benthamiana, N. glutinosa, Chenopodium quinoa* and *C. amaranticolor*. After five weeks there was no visible difference between plants inoculated with extract from lyophilised leaf tissue and mock-inoculated control plants. *N. benthamiana* plants inoculated with fresh orchid leaf tissue exhibited mild to severe non-necrotic symptoms typical of virus infection, including leaf distortion, stunting, and mosaic patterns on the leaves. Other experimental host species inoculated with fresh leaf extract showed no symptoms after five weeks. RNA extracted from newly emerged leaves of inoculated plants was analysed by RT-PCR using primer pairs DOSV4F5200, DOSV4R6100 and DOSV8F6800, DOSV8R7700 (Table S1). RNA extracted from lyophilised leaf of plant Di4 from which isolate Mariginiup11 was identified was used as a positive control for RT-PCR assays. Amplicons of the expected size of ~900 nt were obtained from newly emerged leaf material of symptomatic *N. benthamiana* plants. Upon Sanger sequencing of the amplicons, sequences matched the DOSV genome sequences in the regions expected, confirming that the virus replicated in them. Thus, the process of lyophilization inactivated the virus, although RT-PCR amplicons could be generated from it. 

### Virus distribution amongst wild orchids

A limited survey of wild orchids was done to assess distribution of the virus. RNA was extracted from 144 *D. longifolia* and 102 plants of *C. latifolia* plants from Caporn Park in groups of 10 or 11 leaves and analysed by RT-PCR using two primer pairs (DOSV8F5200 and DOSV8R6100; DOSV10F6800 and DOSV10R7700). No amplification products were detected from those *D. longifolia* samples, but one group of 10 *C. latifolia* leaves yielded amplicons for both primer pairs. When the amplicons were sequenced in both directions using the primers that amplified them, the nt sequences were 98.7 % identical (DOSV8F5200 and DOSV8R6100) and 99.2 % identical (DOSV10F6800 and DOSV10R7700) to the homologous sequence of isolate Mariginiup11 (data not shown). A further survey of 89 *D. longifolia* and 27 *C. latifolia* plants collected from remnant bushland located on Murdoch University campus, Perth, about 47 km south of Caporn Park, failed to identify the virus.

## Discussion

Complete and near-complete genome sequences of two strains of a previously undescribed plant virus, provisionally named Donkey orchid symptomless virus (DOSV), were determined from two asymptomatic wild donkey orchid plants, and detected by RT-PCR in pink fairy orchids. Like the Australian monotreme the platypus [22], DOSV is made of apparently disparate parts. The 69-kDa protein, replicase and CP resemble homologues found in alphaflexiviruses and tymoviruses from plants, the MP resembles MPs found in tombus-like plant viruses, the 44-kDa and the 14-kDa proteins may have distant links to groups of viruses that infect animals, and the 27-kDa protein is unlike proteins described from any source. 

### Wild plant viruses

The considerable genetic deviation of DOSV from described viruses, and its asymptomatic presence in orchids indigenous to Western Australia is evidence that this virus probably evolved in the region. Ancient and persistent associations between host and viruses often do not induce visible symptoms of infection, whereas recent ones usually induce acute symptoms [2]. Recent studies of wild and captive populations of four donkey orchid species from Australia showed that a number of exotic and indigenous viruses infect plants, and in most cases, exotic virus infection induced acute symptoms of infection on the host, whereas indigenous viruses infected without obvious symptoms [6]. Although we consider it unlikely, it is possible that this virus evolved outside Australia and was transported to Australia in exotic plants or vectors during the approximately 185 years since colonization by Europeans. 

Until recently there was limited interest in virus ecology in wild systems, but this is changing [1,3,23,24,25,26]. Studying viruses in natural systems is important for a number of reasons, not least because wild plants are the ultimate sources of viruses that cause epidemics in cultivated systems. Also, evidence points to some wild plant-virus relationships playing roles in tolerance to heat and drought stress [27,28]. Study of mutualistic relationships between plants and viruses may have application to agronomy as climatic extremes occur more regularly. As greater numbers of new types of viruses are identified in wild systems by high-throughput sequencing technologies, it is important that research is carried out into the roles that these new viruses play in ecosystem dynamics [29,30,31].

### Abundance

DOSV occurred uncommonly; it was detected in only two individuals of the 264 *D. longifolia* plants tested, and in one mixed group of 10 plants of the 129 *C. latifolia* plants tested from two sites. It may be an uncommon species, perhaps because it is spread inefficiently between hosts, or because its vector is uncommon. Neither of the host species that DOSV was identified from is considered threatened, nor are they uncommon at Caporn Park. Equally, its apparent rarity could be a function of the quite low number of plants tested, the location of the collection site in relation to its natural distribution, or its primary host is another untested species. Its presence in some, but not all the common donkey orchid plants tested, indicates that DOSV is probably not vertically-transmitted. That it was successfully inoculated to *N. benthamiana* plants under laboratory conditions confirms that DOSV is transmissible. Ongoing transmission experiments should utilize fresh leaf material because lyophilization apparently deactivates the virus.

### Classification

Within the virus order *Tymovirales* is the plant and fungi-infecting family *Alphaflexiviridae*, the plant–infecting family *Betaflexiviridae*, the fungi-infecting family *Gammaflexiviridae*, all with flexuous virions, and the plant-infecting family *Tymoviridae*, that has icosahedral virions. Members of the *Tymovirales* have distinctive replicases that fall into two closely-related lineages, the *Potexvirus*-like (*Alphaflexiviridae*, *Gammaflexiviridae*, *Tymoviridae*) and the *Carlavirus*-like (*Betaflexiviridae*) [32,33,34]. The DOSV replicase is clearly *Potexvirus*-like (Figure 2a), and inclusion of DOSV within the order *Tymovirales* may be considered.

The coat proteins of members of the order *Tymovirales* fall into two groups that do not correlate closely with the replicase groups [32,33,34]. The DOSV CP is clearly related to members of the genus *Allexivirus* (Figure 2b), a group of mainly *Allium*-infecting species, although *Blackberry virus E* (genus unassigned) from a dicotyledonous host shares substantial identity with them [35]. The relatively close identity is suggestive that the virion of DOSV is a flexuous rod like those of flexiviruses, not the icosahedron of the tymoviruses [36]. 

Members of existing families within the *Tymovirales* have MPs that are either of the triple gene block (TGB) type [37] found in all alphaflexiviruses and some of the betaflexiviruses, the 30-kDa protein type [38] found in the remaining betaflexiviruses, or the single tymovirid type that is found in some, but not all tymovirids (some lack MPs) [32]. The DOSV MP is unrelated to those currently described from either the flexivirids or the tymovirids. Instead, it is closest to the 3A-like MPs of viruses found within the genera *Dianthovirus* (family *Tombusviridae*) and *Furovirus* (family *Virgaviridae*), and more distantly to members of the *Bromoviridae* (Figure 3). The position of the CP gene upstream from the MP gene in the DOSV genome is the reverse of the order seen in existing members of the *Tymovirales*. Although the CP of DOSV shares no sequence identity with members of the genus *Tombusvirus*, its position relative to the MP resembles that of *Tomato bushy stunt virus* [39], where the CP also occurs upstream of the MP (Figure 1). 

The 69-kDa protein gene located at the 5’ end of the DOSV genome shares high sequence identity over short regions with the 69-kDa MP located at the 5’ end of the Turnip yellow mosaic virus (TYMV) (genus *Tymovirus*) genome [32], and this is suggestive that they may share similar functions. However, DOSV already has an MP-like gene located at the 3’ terminus of the genome. Existing members of the *Tymovirales* have zero, one, two or three genes involved in viral movement, but so far none have been identified with two MP genes located at opposite ends of the genome. 

Support for inclusion within the order *Tymovirales* is the identity and structure of the replicase, which is closest to *Potexvirus*-like replicases. Its classification there is further supported by the CP, which has identity with those of allexiviruses. Its lower order classification is more problematic because the other DOSV genes are distantly related to genes of other viruses, and there is no precedence for their co-occurrence in one genome. It is envisaged that new lower order taxa will need to be created to classify DOSV.

Recently, new viruses belonging to the genera *Capillovirus* and *Trichovirus* within the *Betaflexiviridae* (order *Tymovirales*) were described from wild orchids and other indigenous plant species in Western Australia [4,5,6,40], and there is evidence that some tymoviruses evolved in Australia [41,42]. Together these findings suggest that viruses within the *Tymovirales* have a long history in Australia. The presence of DOSV further supports this hypothesis. 

## Supporting Information

Figure S1 **Secondary structure of 3’ untranslated region.** Optimal secondary structure of the 3’ untranslated region of Donkey orchid symptomless virus isolate Mariginiup11. The structure shown was predicted in Geneious v6.1.5 and is calculated to have a minimum free energy of -46.70 kcal/mol at 37°C as calculated by the Turner (2004) RNA energy model. Every 20^th^ nucleotide is numbered.
(TIF)

Table S1 **Primer sequences.** Primer pairs used to amplify and sequence the genome of Donkey orchid symptomless virus isolate Mariginiup11. Primers with the same number following ‘DOSV’ are pairs (F = forward primer, R = reverse primer. The numbers following F or R refer to the approximate annealing position on the DOSV genome.
(DOCX)
