# Supplementary figures and images for: Donkey Orchid Symptomless Virus: A Viral ‘Platypus’ from Australian Terrestrial Orchids
Source: PLoS One. 2013 Nov 5;8(11):e79587. doi: 10.1371/journal.pone.0079587 (PMC3818234; doi:10.1371/journal.pone.0079587)

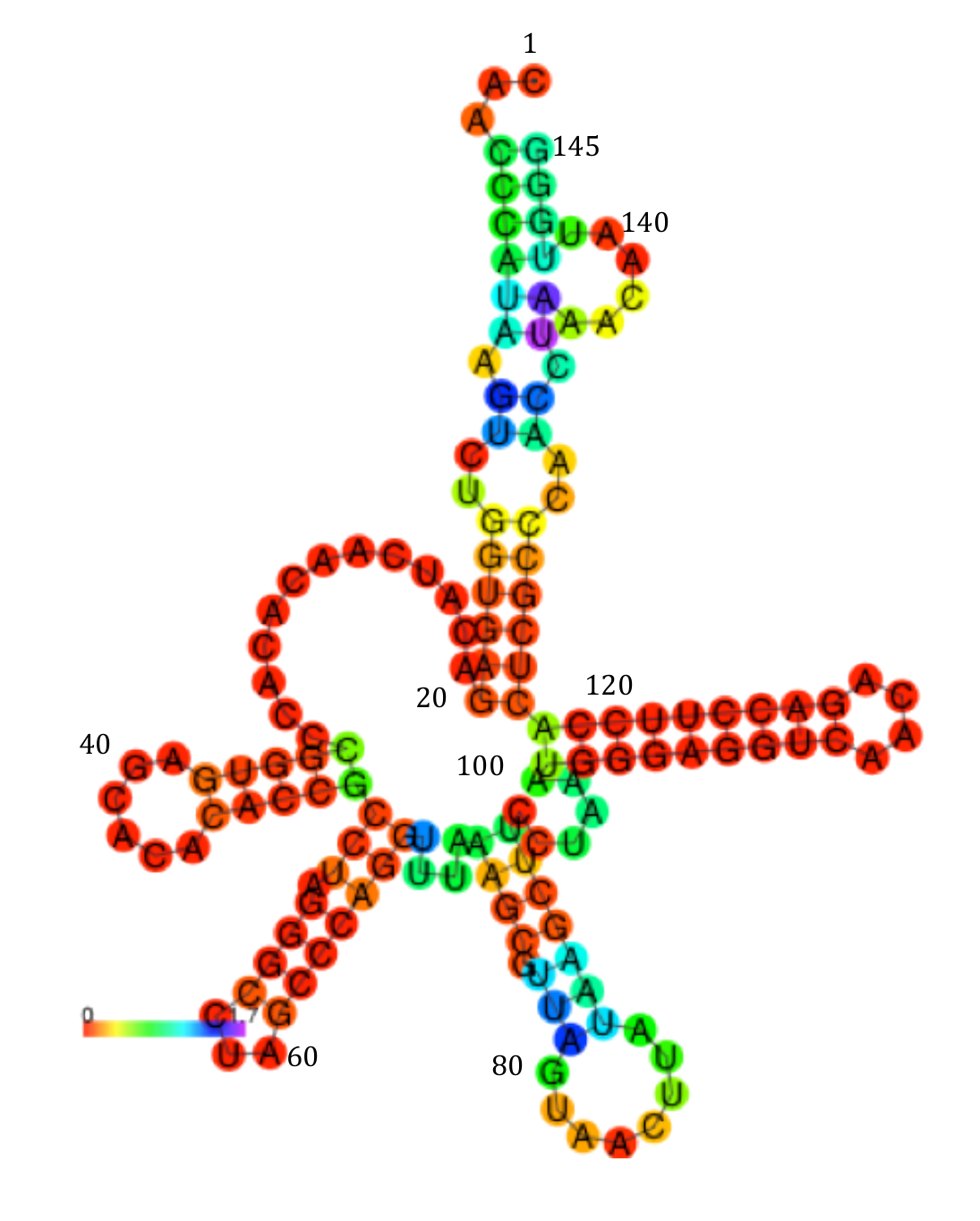

Supplement: Figure S1 — Secondary structure of 3’ untranslated region. Optimal secondary structure of the 3’ untranslated region of Donkey orchid symptomless virus isolate Mariginiup11. The structure shown was predicted in Geneious v6.1.5 and is calculated to have a minimum free energy of -46.70 kcal/mol at 37°C as calculated by the Turner (2004) RNA energy model. Every 20th nucleotide is numbered. (TIF) [file pone.0079587.s001.tif]
