# Supplementary material for: Donkey Orchid Symptomless Virus: A Viral ‘Platypus’ from Australian Terrestrial Orchids
Source: PLoS One. 2013 Nov 5;8(11):e79587. doi: 10.1371/journal.pone.0079587 (PMC3818234; doi:10.1371/journal.pone.0079587)
Supplement: Table S1 — Primer sequences. Primer pairs used to amplify and sequence the genome of Donkey orchid symptomless virus isolate Mariginiup11. Primers with the same number following ‘DOSV’ are pairs (F = forward primer, R = reverse primer. The numbers following F or R refer to the approximate annealing position on the DOSV genome. (DOCX) [file pone.0079587.s002.docx]

| Primer name | Primer sequence 5’-3’ | Calculated Tm ^o^C |
| --- | --- | --- |
| DOSV1F1 | CCA ACC CAT ACT ACT TCG ACG | 61.3 |
| DOSV1R800 | AAG GCA GGC AAG GCC TTG GGT G | 67.7 |
| DOSV2F700 | ACC CTA GCC TAT ACA CCA TCG AG | 64.7 |
| DOSV2R1500 | GCG GTA CAT CTG GTT CGC GTC TG | 68.2 |
| DOSV3F1400 | CGA CAC AAC TGA TCT CTC CCG CG | 68.2 |
| DOSV3R2200 | GCG GCG CGA TGA GTG AGG GCC | 71.1 |
| DOSV4F2100 | GTA GTC GTG CCC ACC AAA GAA C | 64.0 |
| DOSV4R2900 | CTC GGC GAC TTC GTC ATC AAG G | 65.9 |
| DOSV5F2800 | AGC ATG CAC CGA CCG ATC CAG | 65.3 |
| DOSV5R3700 | CCT TCG CCG GTG AGG CGC ATG | 69.2 |
| DOSV6F3600 | CTT GGG GTG CCC GCG CAT GAG | 69.2 |
| DOSV6R4500 | CCG TTC TGG AAG TGC GCG ACC | 67.2 |
| DOSV7F4400 | TTT CAG ATG GCC ATC ATC AAT C | 60.4 |
| DOSV7R5300 | ACA GAT GAT TGA CGC CAA GCG C | 64.0 |
| DOSV8F5200 | TGT CGA AGT TGC AGA GCT CAA AG | 62.9 |
| DOSV8R6100 | TCG ATG AGG GCG TAG GCG TTC | 65.3 |
| DOSV9F6000 | ACA TAT AGT GCT GCC GCC CCC G | 67.7 |
| DOSV9R6900 | AGC GGC AGT GAA TGT TGT TGG | 61.3 |
| DOSV10F6800 | CAT ACC GAA CCT CCA AAC CG | 60.5 |
| DOSV10R7700 | ATG GGT TGT CAA CCT TCG ATG AC | 62.9 |
| DOSV11F7600 | ACC GTC CTT GAA ACC GAC GCT G | 65.9 |
